# Overview of extracellular vesicles in pathogens with special focus on human extracellular protozoan parasites

**DOI:** 10.1590/0074-02760240073

**Published:** 2024-09-23

**Authors:** Johan Alvarado-Ocampo, Elizabeth Abrahams-Sandí, Lissette Retana-Moreira

**Affiliations:** 1 Universidad de Costa Rica, Facultad de Microbiología, Centro de Investigación en Enfermedades Tropicales, San José, Costa Rica; 2 Universidad de Costa Rica, Facultad de Microbiología, Departamento de Parasitología, San José, Costa Rica

**Keywords:** extracellular vesicles, protozoa, parasites, infectious diseases, arthropods, helminths

## Abstract

Extracellular vesicles (EVs) are lipid-bilayered membrane-delimited particles secreted by almost any cell type, involved in different functions according to the cell of origin and its state. From these, cell to cell communication, pathogen-host interactions and modulation of the immune response have been widely studied. Moreover, these vesicles could be employed for diagnostic and therapeutic purposes, including infections produced by pathogens of diverse types; regarding parasites, the secretion, characterisation, and roles of EVs have been studied in particular cases. Moreover, the heterogeneity of EVs presents challenges at every stage of studies, which motivates research in this area. In this review, we summarise some aspects related to the secretion and roles of EVs from several groups of pathogens, with special focus on the most recent research regarding EVs secreted by extracellular protozoan parasites.

Research in cell biology has been developing for years to generate structural and functional descriptions of fundamental mechanisms for life, accompanied by molecular biology techniques. The application of this knowledge in the biomedical field has been renewed with the establishment of an investigation line around extracellular vesicles (EVs). In general, EVs in mammals are described as vesicular bodies that are released from the cell through, at least, two pathways: fusion of multivesicular endosomes (MVE) with the plasma membrane (exosomes; diameter: 50 - ~ 200 nm) or direct budding from the plasma membrane (ectosomes; diameter up to 1000 nm);^1^ ^,^ ^2^ however, it is impossible to claim that fractions contain only one type of vesicle.^3^ The most studied machinery for EVs generation is the endosomal sorting complex required for transport (ESCRT) subcomplexes system, a highly conserved assembly in mammals; homolog proteins and processes have been described also in protozoa.^4^ The biogenesis process will influence the vesicle’s content (or cargo) which, in turn, also depends on the cell of origin and the cellular microenvironment and state. In general terms, proteins, lipids, DNA, and different types of RNAs are part of the EVs cargoes.^5^

The composition of EVs and, fundamentally, their proteome, is defined for the first instance by those proteins involved in their formation machinery, such as Tsg101 and Alix; in addition, proteins of the tetraspanin family (CD63, CD9, CD81) are frequently found in these vesicles and in apoptotic bodies, also considered EVs (but not addressed in this work). Other structural components are cytoskeletal proteins and glycoproteins.^6^ ^,^ ^7^ However, it is important to highlight that this general composition could vary depending on the cell of origin, so that characterisation analyses to evaluate the composition of EVs secreted by a specific type of cell or organism should be performed since there is a lack of universal identification methods for EVs.^8^

The biological role of EVs has been understood mainly as the effects they exert on acceptor cells: (i) as part of intercellular communication, through the transport of active molecules, (ii) and results on immune response and tissue repair/regeneration processes.^9^ Likewise, one of the major areas of global interest for the study of EVs is tumour biology, where cancer cell derived EVs have been found to fulfil a range of functions, from supporting tumour progression, angiogenesis and development of the tumour microenvironment to, conversely, anti-tumour effects from EVs of cancerous and non-cancerous origin mediated by the immune response, reflecting their heterogeneity and the need for further research.^10^ ^,^ ^11^ However, in view of the description and characterisation of EVs in these pathologic conditions and various biological fluids, the idea of developing a “liquid biopsy”’ has been supported thanks to exosomal markers, and it would allow not only detection but also prognosis.^12^ Thus, EVs have become interesting candidates: (i) for the identification of biomarkers associated with different pathological processes, (ii) as delivery platforms with EVs subjected to bioengineering procedures and iii) cell-free candidate vaccines.^13^

Although the clinical utility of EVs in understanding and approaching malignancies such as cancer has been of great interest, their role in infectious processes is another line to be exploited as they are involved as messengers of the immune response and inflammatory processes, with cytokine stimulation, antigen presentation through major histocompatibility complex (MHC)-I and MHC-II, and activation of T and B cells,^14^ as well as their ability to act as key molecule carriers and potential cellular intercommunicators.^15^ The isolation of EVs has been carried out from multiple pathogenic microorganisms (PEVs) and their composition and activity is varied from case to case, even within the same taxonomic genus, although the animal model, the experimental design and the cell type assayed should be associated with this variation.^14^ In this sense, a variety of reviews addressing EVs from pathogens have been published.^16^ ^,^ ^17^ ^,^ ^18^ ^,^ ^19^

Microbial pathogenesis is also a studied process in which PEVs participate by harbouring genes and virulence factors, toxins and molecules for coordination and communication between pathogens.^20^ PEVs also have a role in the pathogen-host interplay, by manipulating or interfering with the immune response or cellular specific cascades due to RNA signalling molecules, protein ligands or pathogen-associated molecular patterns (PAMPS).^21^ ^,^ ^22^ ^,^ ^23^ On the other hand, many of these organisms (bacteria, fungi, parasites, viruses) have also completely intracellular stages, phases, or life cycles in which they can hijack the endosomal machinery of the invaded cells to modify or alter EVs trafficking, induced, in this case, by the pathogen.^24^

The group of medically important parasites comprises agents, mainly protozoans, with complex life cycles, involving invasive and non-invasive evolutionary stages. The role of extracellular vesicles in influencing parasitosis could have several edges: from functions of adaptation to the host environment and effects on the pathogen’s infectivity, to involvement in invasion signalling and immune modulation.^25^ In this review, a brief overview of the main findings in the EVs field, in relation to parasites (with special attention to extracellular protozoa) is presented.

EVs in microbiology

During infections, EVs have not fully deciphered or described roles, and the extent to which they contribute to pathogen establishment is a topic to be exploited in different areas of microbiology and cell physiology. In microbiology, the secretion of EVs from some types of viruses, bacteria, fungi, and parasites has been described and extensively studied in some cases. As it has been reported elsewhere and it’s not the main objective of the review, only a brief description of highlights regarding EVs of non-parasitic origin will be presented.

EVs can be aroused from virus-infected cells. In these cases, EVs can also carry viral elements, such as proteins or receptors that make the acceptor cell more susceptible to infection, as described for human immunodeficiency virus (HIV),^26^ ^,^ ^27^ and similar to the transference of CD9 and ACE2 receptors that has been recently proposed for severe acute respiratory syndrome coronavirus 2 (SARS-CoV-2), the causative agent of coronavirus disease 2019 (COVID-19).^28^ In addition, viruses such as hepatitis C can use EVs as machinery to infect cells via viral RNA and achieve replication without relying on the virion or viral receptors.^29^ ^,^ ^30^ In this sense, it could be suggested a dual functionality, as they also represent a means to trigger antiviral responses by the activation of adaptive immunity via viral antigens and molecular effectors. However, a major methodological limitation is the complexity involved in separating viral particles from the EVs to be assayed.^18^

EVs of prokaryotic origin have been found not only in *in vitro* cultures, but also in *in vivo* cultures and even from environmental samples.^31^ ^,^ ^32^ These are essentially the same bilayered particle as in eukaryotic cells, as they correspond to membranous “capsules” released by a cell into the extracellular space, but there are fundamental structural differences given by the conformation of the cell envelope. For instance, in gram-negative bacteria, EVs are rich in lipopolysaccharides, although there are generally cargoes common to all bacteria such as proteins involved in metabolic pathways and genetic material.^17^ ^,^ ^33^ In bacteria, the formation and secretion of the so-called outer membrane vesicles (OMVs) is given globally by gene regulation^34^ ^,^ ^35^ and different approaches such as: (i) a particular distribution of phospholipids in a membrane region, or (ii) the accumulation of molecules in the periplasmic space and the consequent turgor pressure and interaction between negative charges, which promote plasma membrane’s curvature.^36^ ^,^ ^37^ Likewise, the release could be related to compromises in membrane stability, where there are lytic effectors leading to disruption of the peptidoglycan wall.^38^ ^,^ ^39^

Regarding their physiology and clinical relevance, OMVs have been involved not only in bacterial communication processes, horizontal gene transfer and influence on the microenvironment,^17^ but also their cargo has been employed as an arsenal to interact with the host; in fact, TLRs have been involved in interactions of EVs with mammalian target cells^40^ ^,^ ^41^ and their content may include virulence factors with cytotoxic and antibiotic resistance effect.^42^ ^,^ ^43^ ^,^ ^44^ On the other hand, regulation of signalling pathways leading to immunomodulation has been determined in bacteria of the oral and intestinal microbiota.^45^ A recent study with cervicovaginal pathobionts and commensal bacteria EVs indicate differential cargo and viability/cytoadherence effects when evaluating them onto a culture model with ectocervical cells and *Trichomonas vaginalis*, showing a possible role in host-pathogen interaction.^46^

The study of EVs in this type of pathogens has been virtuous: *Escherichia coli*, *Moraxella catarrhalis* or *Pseudomonas* spp. have been subjects of research, as well as the gram-positive *Bacillus subtilis* and *Staphylococcus aureus.* ^47^ Actually, licensed OMVs based vaccines against meningococcal infections have been developed and, in this sense, advanced discovery is currently exploring on enteric pathogens.^48^ Besides, and thanks to electron microscopy, EVs have also been described in mycobacteria and fungi of medical importance.^47^ Particularly, in the latter, it should be noted that their secretion has been described in yeasts such as *Cryptococcus neoformans*,^49^ as well as in filamentous fungi such as *Sporothrix brasiliensis*.^50^

EVs in fungi, as eukaryotic organisms, share release mechanisms with those described in mammals, as they appear to be linked to the endocytic secretory pathways associated with the ESCRT and Golgi reassembly and stacking proteins (GRASP) machinery, and the ER-GA-exocyst-PM axis.^51^ Likewise, their size ranges from 20 - 50 nm to 1000 nm, according to different reports and under different methodologies such as dynamic light scattering (DLS), electron microscopy (EM) and nanoparticle tracking analysis (NTA).^16^ Other vesicles studied in this group have been the periplasmic vesicles, which are those inside the fungal cell wall, between the cell membrane and the inner face of the chitin barrier,^51^ but also EVs from protoplasmic models have helped to understand fungal vesicles roles.^52^ Finally, in addition to pathogenic related functions, cell wall-remodelling enzymes for easier vesicle passage and immunogenic protein content have been found in EVs of *Histoplasma caspsulatum*;^53^ also, fungal EVs are suspected to be involved in modulation of immune effectors and in cryptococcosis and sporotrichosis outcome due to virulence enhancement.^54^

Important findings in EVs for clinical parasitology

The release of EVs in the context of a parasitic infection becomes complex as one has those produced by the host and by the parasite.^14^ In fact, it has been proposed that the fusion between the EVs of protozoan parasites and those of the host cell could have effects on any of the involved; that’s why the understanding the participation of EVs in host-parasite interaction and cell communication would probably redefine the concepts of parasitism.^55^ Furthermore, the dissemination of genetic elements of the parasite through EVs supports their possible involvement in co-adaptative and co-evolutionary processes of gene regulation and synchronisation with the host metabolism.^56^ The role as strong parasite-parasite communication messengers and further effects on this regard, as motility/migration signals in African trypanosomes has been demonstrated,^57^ cargo manipulation and functional small RNA roles in PEVs are advancing areas in this research field.^58^

The proteome and transcriptome of parasitic EVs reveals the presence of molecules associated not only to immunomodulation, but also to reproduction and survival, so that any subsequent discovery in relation to their functions would give rise to new ways of understanding pathogenesis, how parasite-host communication occurs, and the study of new drug targets.^59^ Even diagnostic applications are already on the horizon, as proposed by Wang et al.,^60^ who worked in the development of a biosensor to discriminate between EVs from *Ascaris suum* and those from mice macrophages, where the differential binding of these EVs through a specific marker (CD63), absent in the parasite EVs, causes a shift in the wavelength resonance;^60^ even though, each potential diagnostic tool should be carefully validated due to, for example, the possibility of finding other tetraspanins in parasite-derived EVs.^61^ ^,^ ^62^ For this reason, the proper characterisation of the content of EVs is relevant.

During the different forms of parasitism, PEVs derived from extracellular parasites could be found, but also those secreted from intracellular infected cells and parasitic antigen stimulated cells.^63^

Macro-extracellular parasites: arthropods and helminths

Before delving into protozoan parasites, it is worthwhile to review what has been identified in other groups of classical parasitology, such as arthropods that act as biological vectors and helminths of medical importance, with specific cases of their EVs pivot findings.

EVs of some arthropods have been implicated in the dissemination/infection process of the microorganisms they transmit,^64^ but also as part of the vector-host-pathogen triad.^65^ In this sense, these vesicles have been described as possible mediators in the transmission of flavivirus proteins and RNA, as demonstrated by Zhou et al.^66^ in their *in vitro* model with an *Ixodes scapularis* cell line infected with langat virus (LGTV) and human keratinocytes/endothelial cells. The same was demonstrated in cell lines derived from *Aedes aegypti* and *Ae. albopictus* mosquitoes with dengue virus type 2 (DENV2) viral particles.^67^ Besides, viral-like particles have been observed in extracellular vesicles derived from DENV infected C6/36 cells.^68^

In addition, Oliva Chávez et al.^69^ demonstrated an impaired feeding ability of *I. scapularis* by silencing genes of soluble NSF (N-ethylmaleimide-sensitive fusion protein) receptor (SNARE) molecules (vamp33 and synaptobrevin 2) related to the release of EVs, in parallel to an increase of γδ-T cells at the site of the bite. In turn, these EVs present in tick saliva might play roles not only in tick-borne pathogens transmission dynamics, as it has been seen for protozoan parasites and bacteria, but also in feeding-facilitating immunomodulatory responses at the ectoparasite-host skin interplay.^70^ Moreover, the vector as an arthropod host could be affected by microbial EVs, as the case of the regulation of the innate immune response of *Ae. aegypti* by EVs of microfilariae.^71^ Of course, some non-vector free living arthropods like dust mites have been implicated in other types of human damage such as allergic processes and, for instance, *Dermatophagoides farinae* EVs were shown to be immunoreactive against specific serum IgE and to induce airway inflammation in mice.^72^

In the helminths group, EVs, as part of the excretory-secretory products, have been studied from different perspectives, such as in *Trichuris muris*, for pathogenesis understanding purposes using organoids,^73^ or in *Fasciola hepatica* and *Brugia malayi*, where proteomics and inmunological-based visualisation techniques have been instrumentalised to elucidate the biogenic pathways and cellular origin of vesicles.^74^ ^,^ ^75^ The participation of carbohydrates in lectin-EVs binding patterns and macrophage internalisation has also been evidenced,^75^ as well as the description of virulence factors in EVs from *Paragonimus kellicotti* lung cyst fluid^76^ and *Echinococcus multilocularis* protoescoleces.^77^ Moreover, varied functional-immune assays have revealed the immunomodulatory capacity of *Trichinella spiralis* EVs,^78^ the phenotypic modification of dendritic cells and the reduction of macrophages migratory capacity induced by EVs from the trematode *F. hepatica* ^79^ ^,^ ^80^ and the expression of miRNAs associated with the mTOR signalling pathway as part of the cargo in EVs from filarial nematodes,^81^ which all supports a potential for downregulation. This type of active biomolecules (miRNAs) derived from EVs are part of the developing diagnostic arsenal, as they could be identified from biological samples and have been achieved from serum of *Schistosoma* spp. infected patients.^82^ Computational prediction of miARN found in EVs from different nematodes support their immunological relevance as immune networks genes are targeted by these molecules.^83^ ^,^ ^84^ ^,^ ^85^ For helminthiasis, as in other microorganisms, the production of vaccines based on EVs and their antigens is still an interesting proposal that gives new routes for resolving doubts about antigen expression control and its variability, the response that can be induced or the adjuvants to be used.^86^ In mice immunised with *F. gigantica* exosome-like particles, burden reduction after metacercariae infection and immunoglobulin production has been pointed out.^87^ On the other hand, immunogenic antigens as part of the cargo of EVs of helminths might be an interesting subject for immunodiagnostic advances research.^88^ ^,^ ^89^

Protozoan parasites

Many protozoan parasites successfully exert intracellular parasitism and have adapted to their human hosts in such a way that they are even able to modulate, at a certain stage, part of the interaction with the vascular endothelium and its microenvironment through EVs, facilitating the establishment of infections such as it occurs in malaria.^90^ ^,^ ^91^ Likewise, infected host cells can induce pro- and anti-invasion responses through their EVs.^92^ However, the first contact between the parasite (sometimes coming from a vector) and the host tissues necessarily occurs in invasive forms that, to continue the life cycle, eventually reappear at certain times or under specific circumstances.^93^ ^,^ ^94^ In the framework of experimentation with protozoa and their EVs, these vesicles can sometimes be studied in axenic culture.^94^

Intracellular protozoan parasites

In apicomplexan-related infections like malaria, provoked by species of the obligate intracellular parasite *Plasmodium*, the study of EVs obtained from its invasive stage is scarce, since the growth of the parasite requires the use of cell culture. In this sense, several works have focused on the study of infected-red cell derived EVs;^95^ ^,^ ^96^ however, it would be worth exploring the role of EVs from their sporozoites and merozoites in the mechanisms of invasion of hepatocytes and red blood cells.

EVs secreted by tachyzoites of *Toxoplasma gondii*, another parasite, have also been characterised using transmission electron microscopy (TEM) and NTA, and purified by gel exclusion chromatography.^97^ ^,^ ^98^ Furthermore, they have been related to: (i) the *in vitro* stimulation of a proinflammatory profile in macrophages,^97^ (ii) the expression of different miRNAs as possible cargoes,^98^ ^,^ ^99^ (iii) the promotion of host immune evasion,^100^ and (iv) enhanced virulence (in terms of parasitaemia) in mice, five days post infection (p.i.) (through co-inoculation of EVs and tachyzoites).^98^ Immunisation with tachyzoite-released EVs showed to trigger humoral immune responses, increasing the survival rate of mice challenged with a lethal dose of parasites. Finally, immunohistochemistry showed high expression of tumour necrosis factor (TNF-α) in spleen cells, along with IL-10 and interferon (IFN-γ) in spleen and brain cells.^101^

On the other hand, the trypanosomatid protozoan parasites *Trypanosoma cruzi* and *Leishmania* sp., which cause American trypanosomiasis (Chagas disease) and leishmaniasis, share the characteristic of being transmitted to humans mainly by arthropod vectors: triatomine bugs and sandflies, respectively. The effect of EVs in the interaction of these parasites with their vectors during the extrinsic cycle (stage in which they also manifest themselves extracellularly) has been catalogued as negative for early migration of *T. cruzi* in the digestive tract of *Rhodnius prolixus* pre-fed with epimastigote-derived EVs; although with no effect on the amount of metacyclic trypomastigotes (the infective form for humans) at 28 days p.i., nor in *Triatoma infestans* in general.^102^ The secretion of parasite EVs occurs not only in the arthropod midgut, but also at the vector-host interface, as it has been demonstrated with *Leishmania*-derived EVs present in the inoculum at the site of the bite.^103^

The first encounter of *Leishmania* sp. with host cells occurs at the dermal level.^104^ Mice footpad co-injection of EVs and metacyclic promastigotes of *L. major* causes exacerbated swelling and increased parasite load, with a rise in the expression of proinflammatory cytokines such as IL-17a.^103^ ^,^ ^105^ In counterbalance, the production of IL-6 and IL-10, along with the de-stimulation of TNF-α, has also been observed in monocytes and macrophages in the presence of *Leishmania-*derived EVs,^105^ ^,^ ^106^ associated with an immunosuppressive effect and benefiting parasite’s survival.^107^ Indeed, the presence of GP63 in *Leishmania* EVs represents an anti-inflammatory regulation mechanism.^108^ Besides, an important enrichment of RNA cargo has been found in 120 nm EVs of axenic cultures of *Leishmania*.^109^

Back to the case of *T. cruzi*, the causative agent of Chagas disease, in the context of the acute phase of the infection, there are several interesting findings: EVs produced during early parasite-host contact promote parasite infectivity in Vero cells^110^ ^,^ ^111^ and their injection in mice prior to trypomastigote inoculation leads to more inflammation, higher parasitism and formation of amastigotes nests, with CD4+ lymphocytes infiltration in the heart.^112^ It has also been proven that *T. cruzi* EVs can inhibit complement lytic activity,^113^ which is a form of initial immune evasion.

More recent studies on *T. cruzi* trypomastigote-derived EVs reveal an increase in Ca^2+^ mobilisation and permeabilisation in Vero cells treated with these vesicles,^111^ as well as the induction of a proinflammatory profile of cytokines (TNF-α and IL-6) in macrophages and muscle cells.^114^ EVs in *T. cruzi* may diverge in structure and composition, depending on the stage of the parasite (trypomastigote vs. epimastigote):^115^ average sizes of 183 nm and 259 nm were determined in epimastigote-derived EVs, resulting larger than trypomastigote-derived EVs (60 nm and 143 nm). Moreover, significant differences were found in the exoproteome, particularly in one of the most important virulence factors: proteins of the trans-sialidase family, with greater presence and diversity in trypomastigote-derived EVs.^115^

Even though the specific generation pathways of EVs in trypanosomatids are unknown,^94^ there is some evidence that there could be ESCRT independent mechanisms, as nanotube derived EVs in *T. brucei*, ESCRT dependent multivesicular bodies (MVBs) in *Leishmania*, or new biogenesis pathways like reservosomes EVs in *T. cruzi*.^4^ Cargoes of trypanosomatid-derived EVs are the reflection of well-known glycoproteins and soluble proteins from the parasite, which eventually interact with TLRs.^116^ Besides, an interesting approach for implication in virulence might be related to the capacity of EVs of inducing/transferring resistant phenotypes or improving parasite fitness.^117^ ^,^ ^118^

Other trypanosomatid, already mentioned, but eminently extracellular along its life cycle is *T. brucei*, the causative agent of African sleeping sickness, whose EVs covered with variant surface glycoproteins (VSGs) have been involved in pathogenesis due to their fusogenic capacity with erythrocytes.^119^ Furthermore, upregulation triggering effects on CD4+ and CD8+ T cells and stimulation of MHC expression in macrophages have also been observed.^120^

Extracellular protozoan parasites

There is another group of unicellular eukaryotes that exert parasitism extracellularly through vegetative forms, the trophozoites, and lack intracellular evolutionary forms; the mechanisms of pathogenesis in these cases involve some effectors other than the intracellular arsenal. However, EVs have come to light in recent research as molecular mediators of these pathogens. In Figure, a depiction of the principal role of their EVs over several scenarios is shown. A size comparison of EVs obtained from extracellular protozoa, by several techniques such as NTA, TEM, and DLS, is presented in Table. This table also summarises isolation methodologies employed by different groups that investigate EVs from these parasites; most of these methods are recommended and implemented by other protozoan EVs researchers.^121^

**Figure:**
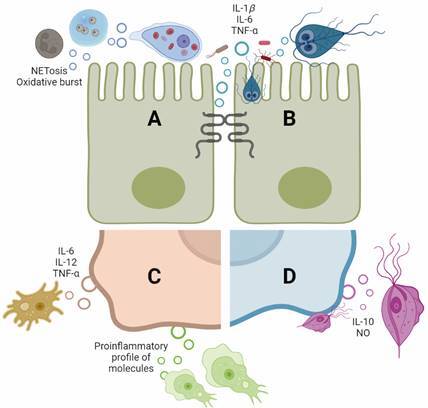
Explored general roles of extracellular vesicles (EVs) derived from extracellular protozoa in pathogenesis, parasite-parasite communication, and its relationship with immune effectors. (A) *Entamoeba histolytica* EVs are possible involved in en/excystment processes and have effects over neutrophils; (B) *Giardia duodenalis* EVs provoke alteration of Caco-2 cells tight junctions and enterobacteria, promote adhesion of the parasite and induce a proinflammatory outcome; (C) free living amoeba (*i.e.*, *Acanthamoeba* sp., *Naegleria fowleri*) derived-EVs are uptaken by glial cells and other mammalian cells and are also associated with a proinflammatory chemokine/cytokine production; (D) *Trichomonas vaginalis* induce the production of nitric oxide (NO) in macrophages and stimulate adhesion of the parasites to ectocervical cells. Figure created with BioRender.com.

*Giardia duodenalis* - *G. duodenalis* (syn. *G. intestinalis*) is an intestinal parasite that adheres, as its trophozoite form, to the epithelium of the small intestine, with effects on enterocytes that induce malabsorptive diarrhoea.^122^ Its cystic form protects it from environmental adversities and differentiation involves the transport of components of the extracellular cystic wall by dense granule-like vesicles, called encystation specific vesicles, whose origin is associated with the ER.^123^ It has been proposed that, during the release of their content, remnants of the plasma membrane catalogued as “empty vesicles or membrane ghosts” are formed and remain attached to flagella or suspended in the extracellular milieu.^124^

During the last decade, EVs have been described as part of the secretome of *G. duodenalis* in axenic cultures,^125^ with average size of 201,6 nm;^126^ now, it is possible to focus on specific size subpopulations. For instance, there is a modified differential centrifugation protocol that enriches populations > 100 nm.^127^ Actually, lipid profiles vary between small and large EVs^128^ which can help to understand to role of some lipid species in EVs release, as it has been proposed,^126^ adhesion, encystation and signalling.^128^ Besides, their involvement in pathogenic processes has begun to be elucidated: there is increased trophozoite adhesion to Caco-2 cells in the presence of *G. duodenalis-*derived EVs, they contribute to the maturation of dendritic cells,^126^ alter tight junctions given by ZO-1 and Claudin-4,^129^ and there are virulence factors such as antigenic variable surface proteins (VSPs) and giardin in cyst-derived EVs^126^ ^,^ ^130^ and trophozoites.^131^ In general, proinflammatory effects and raised immunogenicity are driven by EVs secreted by *G. duodenalis*.^132^

In addition, *G. duodenalis* has also an internal membranous system: peripheral vesicles (PVs), which have been linked to part of the ESCRT machinery,^133^ highlighting the possibility that it operates at this level as part of a secretory pathway. PVs can act as microvesicular bodies with intraluminal vesicles (ILVs), so could be linked to the origin of EVs.^134^ It has been proposed that these occurs in both vegetative and resistance forms, adding a potential link to differentiation between these phases.^134^ Indeed, another author highlights an EVs release that depends on ESCRT-associated molecules.^135^

Among other pathophysiological roles associated with EVs of *G. duodenalis*, a subpopulation of 187,6 nm was able to restore parasite adhesion capacity after the treatment with Cl-amidine, an inhibitor of peptidyl arginine deiminase in Caco-2 cells.^131^ Also, pretreatment of murine macrophages with *G. duodenalis*-derived EVs generated increases in cytokines such as IL-6 and TNF-α, as with it happened with trophozoites.^136^ In addition, *G. duodenalis* EVs induced phosphorylation and activation of p38, ERK and AKT signalling pathways, the NF-κB pathway^136^ and NLRP3 of the inflammasome, which possibly mediates IL-1β production.^130^

Finally, by evaluating the effect of EVs of *G. duodenalis* on commensal bacteria such as *E. coli* and *Enterobacter cloacae*, it was revealed that these vesicles could modulate growth, biofilm formation, motility, and adhesion to the epithelium,^137^ ^,^ ^138^ which suggests new roles in the interaction with host microbiota.

*Trichomonas vaginalis* - Trichomoniasis is the most common non-viral sexually transmitted disease, which mainly affects women in reproductive age, but can also be symptomatic in men.^139^ The parasite causing the disease is the flagellate *T. vaginalis*. In the first description of EVs produced by trophozoites of this agent, an overlapping of protein composition compared to mammalian exosomes was concluded;^140^ this was similar to the findings published by Nievas et al.,^141^ who reported a 56% of proteins homologous to those found in a fraction of human EVs. In addition, most proteins with signalling functions and metabolic enzymes were identified from those with identifiable domains.^140^ Using SEM, an increased protrusion of EVs from parasites due to the presence of CaCl_2_ was shown.^141^

Other cargoes described in *T. vaginalis*-derived EVs are: surface proteins of the BspA family,^140^ ^,^ ^141^ which are molecules possibly involved in pathogenesis; ARF proteins, relevant for their relationship with formation, release and cargo selection;^141^ tetraspanin TSP1^140^ ^,^ ^142^ and VPS32, a molecule involved in the ESCRT III complex, which in *T. vaginalis* is related to the biogenic regulation of EVs, cargo sorting and parasite adhesion;^143^ tRNA fragments^46^ and Trichomonasvirus particles, that might be transmitted to the host and is a possibly a critical element in disease development.^142^ ^,^ ^144^ Proteins involved in filopodia and in the formation of cytonemes (*e.g.*, small actin-binding proteins, calreticulin and Rho/Ras family proteins) were found in EVs implicated in parasite-parasite communication.^145^

Characterisation of the EVs uptake by host cells demonstrated the fusion with ectocervical cell membranes to release their contents^140^ and internalisation, with fluorescence and fluorimetry assays in BPH-1.^146^ This uptake might be Ca^2+^-dependent, mediated by glycosaminoglycans and heparan sulphate in proteoglycans from host cells and 4-α-glucanotransferase homologues that act as ligands in EVs.^146^ Entry by action of caveolin-1 and lipid raft dependent endocytosis has been established,^46^ which has been successfully inhibited by cholesterol depletion agents.^146^

Regarding the pathogenic process, it could be pointed out that EVs (from a high adherent strain) increased adhesion by stimulating both host cells and parasites from less adherent strains;^140^ the same group demonstrated a positive outcome in survival and parasite burden when co-incubated with EVs, confirming a role in colonisation.^147^ Nitric oxide (NO) production in macrophages has been detected, indicating EVs-mediated activation.^148^ When animal and cellular models are pre-treated with EVs of *T. vaginalis*, an immune response has been determined, with a mitigating tendency that reduces mice oedema and inflammation and with significant increases in IL- 10;^148^ conversely, IL-6 is elevated to a lesser extent and no real regulation by EVs has been observed.^140^

*Parasitic and free-living amoebae (FLA)* - In amoebae such as *Dictyostelium discoideum*, EVs were described since 1998, as vesicular organelles of 100 - 300 nm.^149^ This organism has been tested as a eukaryotic model for the study of several diseases, cellular processes, and host-pathogen interactions, due to its easy manipulation and growth.^150^ Therefore, it has also been postulated as a potential model for research on the heterogeneity of EVs and the elucidation of their biological functions.^150^

Subsequently, with the escalate of interest in EVs in different research groups, work lines have been developed around the pathogenic intestinal amoeba such as *Entamoeba histolytica*, but also on free-living organisms with pathogenic potential (amphizoic) such as *Acanthamoeba* sp. and *Naegleria fowleri*, with highlights of the possible intervention of EVs in the mechanisms of damage and pathogenesis. Besides, an emerging issue is related to encapsulation of pathogenic bacteria (such as *Legionella pneumophila*) and respiratory viruses in EVs secreted by FLA, EVs serving as easy alveoli contact spreaders.^151^ ^,^ ^152^

*Entamoeba histolytica* - The large intestine is an ideal habitat for colonisation by amoebae and, particularly, *E. histolytica* has been studied as a potentially invasive agent with complications such as ulcerative colitis and amoebic dysentery.^153^ With the help of proteomic analyses, molecules involved in the pathogenesis of this amoeba have been identified, like adhesins and cysteine proteases; interestingly, membrane recycling has been suggested since surface membrane proteins were also identified in the excretion-secretion products.^154^

Following a study of the endomembrane system, vesicles of 50 - 200 nm were known to be present inside the parasite with possible roles in a protein traffic system together with MVBs and endosomes, as well as the presence of mammalian Alix orthologues in the vesicles,^155^ establishing a possible role of the ESCRT complex. Later, EVs of 125 nm were obtained from axenic culture of *E*. *histolytica*,^156^ and a broader range of sizes (50 to less than 600 nm) has also been shown through TEM and NTA.^157^ Amoebic EVs were enriched in cell surface galactose/N- acetyl galactosamine-binding lectins and an important part of proteins unveiled by mass spectrometry did not present signal peptide; also, selective small RNA packaging was described and compared to cellular RNA, denoted some differences^156^ Packaging of tRNA fragments also occurs.^158^

Functional assays with neutrophils have demonstrated incorporation of amoebic EVs and effects over oxidative burst and NETosis,^157^ and intercommunication between parasites in encystment processes.^156^ The latter was seen in a model using *Entamoeba invadens*.

*Acanthamoeba* sp*.* - Amoebae of the genus *Acanthamoeba* are ubiquitous in nature and capable of generating a central nervous system condition such as amoebic granulomatous encephalitis, but also other more frequent diseases such as amoebic keratitis. The cases are typically associated to genotype T4 and, to a lesser extent, T3 and T11,^159^ among others. In environmental isolates, our research group has described organisms from these and other genotypes with pathogenic potential,^160^ ^,^ ^161^ ^,^ ^162^ including the secretion of EVs with serine and cysteine protease activity in *Acanthamoeba* T5.^160^ Coincidentally, another study found that serine proteases are predominant in four strains of environmental (genotypes T1, T2 and T11) and clinical (genotype T4) origin.^163^ Aminopeptidase activity has also been determined in EVs of *Acanthamoeba*.^164^

A previous study with *Acanthamoeba castellanii* described evaginating vesicles from plasma membrane using SEM, and great diversity in mean diameter estimations (Table): 117 nm by TEM and 287,7 and 365,1 nm using DLS,^165^ a range that embraces sizes reported in posterior works (166,7 nm using NTA).^164^ When analysing two culture conditions through a qualitative proteomic characterisation of the secretome (one in rich medium PYG and the other under nutritional stress), most of the proteins belonged to the miscellaneous or undefined categories.^165^ However, the exoproteome under stress identified more proteins related to cellular stress and oxidative, protein and amino acid metabolism, with a rich enzymatic profile for carbohydrate metabolism (amylases, glycosyl hydrolases, alpha-1,4-glucan phosphorylases),^165^ which draws attention for its potential use in biotechnological applications.^166^ While, in abundance, more locomotion and signalling proteins were found,^165^ other proteomic analyses of quantitative type support that the largest families of proteins found are hydrolases and oxidoreductases.^164^ On the other hand, characterisation of lipid composition has shown the presence of sterols, phospholipids, free fatty acids, and sterol esters.^165^

**TABLE:** Isolation and characterisation methods commonly employed for the collection of extracellular vesicles (EVs) from extracellular protozoan pathogens, including sizes ranges reported in literature

Parasite form	Isolation method	EV diameter (nm)	Technique	Reference
*Trichomonas vaginalis* trophozoites	DC + sucrose gradient	~ 50 - 100	TEM	^140^
	DC	50 - 100	TEM	^148^
	DC	30 - 150	TEM	^142^
	DC	100 - 1000	TEM	^141^
		380 / 63	DLS	
	DC + density gradient	108 - 146	DLS	^144^
DC	~ 105	NTA	^(46)^
*Giardia duodenalis* trophozoites	DC	20 - 25 / 50 - 100	TEM	^(135)^
		22,8 / 85,2	DLS	
	DC	60 - 150	TEM	^(126)^
		150 - 350	NTA	
	DC	100 - ~ 200	TEM	^(130)^
		143,5	NTA	
	DC	50 - 90 / 117 - 282	TEM	^(128)^
		82,6 / 238,5	NTA	
	DC	187,6 / 67,7	NTA	^131^
	ExoEasy maxi kit (QIAGEN)	CaCl_2_ treatment: 210 Bile treatment: 270	NTA	^137^
*Entamoeba histolytica* trophozoites and/or cysts	Total exosome isolation (Invitrogen)	< 200	TEM	^157^
		< 50 - 600 Peak: 483	NTA	
	Total exosome isolation (Invitrogen)	125	NTA	^156^
*Acanthamoeba* sp. trophozoites	DC	PYG medium: 31,9 - 467 Glucosed medium: 33,7 - 303,2	TEM	^165^
		PYG medium: 56,1 - 68,4 / 150,4 - 223,0 / 402,9 - 659,4 Glucosed medium: 173,2 - 234,8 / 585,1 - 746,5	DLS	
	DC	28°C incubation: 184,6 ± 50,80 / 50,29 ± 8,49 37°C incubation: 111,3 ± 19,8	DLS	^160^
	Ultrafiltration: Amicon ultracentrifugation filters (Merck Millipore) + Total exosome isolation (Invitrogen)	Peak: 118	NTA	^164^
	DC	101 - 150 / 151 - 200	NTA	^163^
*Naegleria fowleri* trophozoites	DC	43,88 / 207,95	TEM	^169^
		216 ± 83	NTA	
		227,13 ± 37,98 / 206,29 ± 37,08 / 24,24 ± 9,18	DLS	
	Size exclusion chromatography	22,4 - 955	DLS	^172^
	DC	156,8 ± 13,4 / 141 ± 8,3	NTA	^168^
	DC + Size exclusion chromatography	Overall average of five strains: 152,6	NTA	^170^

When data is not presented as a range, it corresponds to a mean or a NTA peak; DC: differential centrifugation; /: indicates different EV subpopulations.

EVs of *A. castellanii* have been shown to interact with different cell lines such as Chinese hamster ovary (CHO) cells, glioblastoma T98G and rat glial C6 cells, adhering to the membrane and terminating in all cases with their internalisation. Likewise, *in vitro* cytopathic effect assays have yielded positive results.^164^ ^,^ ^165^ It has been further determined that *A. castellanii* EVs are also able to elicit an immune response in THP-1 cells, after detecting the expression and production of IL-6, IL-12^164^ and TNF-α.^163^ In murine macrophages, activation level after the stimulation with EVs of *Acanthamoeba* has been measured through the production of NO, demonstrating that, of those tested, the main receptor is TLR4, followed by TLR2.^163^ Protease inhibitors have exerted a negative effect on both, the concretion of the cytopathic effect,^165^ as well as NO production,^163^ pointing to a preponderant role of these as virulence factors associated with EVs.

*Naegleria fowleri* - The infectious disease given by *N. fowleri*, primary amoebic meningoencephalitis, is a severe fulminant pathology with high mortality rate, in which the amoeba employs contact-dependent (adhesion and phagocytosis) and contact-independent (matrix metalloproteinases and pore-forming proteins) mechanisms to produce brain tissue damage and destruction.^167^

Pathophysiological mechanisms are under constant review and two pioneer investigation groups have confirmed the production of EVs by trophozoites of this amoeba. In this sense, it has been reported cup-shaped vesicles observed via TEM, comprising two subpopulations of 156,8 nm and 141 nm;^168^ a more comprehensive characterisation of these EVs was performed by Retana Moreira et al.,^169^ who measured size through TEM, NTA and DLS, obtaining means ranging from 24,24 nm to 227,13 nm (Table). Z-potential of -12,228 mV was also determined.^169^ Then, clustered release of EVs was reported by Russell et al.^170^ and Retana Moreira et al.^171^

Proteome analysis has found almost half of proteins are still uncharacterised, but also identified actin, Rho GTPases, dehydrogenases, and two potential pathogenesis-related factors: leucin aminopeptidase and fowlerpain (a cysteine protease).^169^ Besides, protease activity of EVs of *N. fowleri* has been found, mainly by serine proteases, although to a lesser extent than the whole trophozoite extract.^169^ Afterwards, Russell et al.^170^ identified 2270 proteins, 150 of which overlapped with Retana Moreira et al.^169^ findings.

Regarding functional analysis, cellular effects of *N. fowleri* EVs have been featured by PKH26-monitored internalisation in the THP-1 monocytic cell line, with no subsequent apoptosis and stimulation of IL-8 gene expression, cytokine that was later identified 48 h and 72 h post-activation of macrophages.^168^ Uptake by other mammalian cells (*e.g.*, Vero, HFF, A549, B103 rat neuroblastoma cells) and other amoebae has been proved via EVs-R18 staining.^170^ A cytokine/chemokine proinflammatory profile was described on BV-2 microglial cells stimulated by *N. fowleri* EVs, showing the possibility of a contact independent immunopathogenic mechanism.^172^

In this sense, our group has just confirmed the induction of diverse effectors (*e.g.*, *iNOS*, *IL-6*, *IL-23*, *TNF-α*, *IL-10*) on primary microglia and BV-2 cells by EVs secreted by trophozoites of two clinic isolates of *Naegleria fowleri*. We also noted morphological changes in cells to an amoeboid-like morphology after the contact with these vesicles. Moreover, specific *N. fowleri* DNA was found in EVs fractions, according to our quantitative polymerase chain reaction (qPCR) results,^171^ a promising finding for diagnostic purposes.

Limitations and future perspectives

There are still many biological questions regarding EVs and their purposes; whether they respond to a stimulus, a selective process or an incidental release must be elucidated.^173^ ^,^ ^174^ In parasites of medical importance, it remains to be clarified if the change in the profile of biomolecules depends on the parasite stage and what mechanisms of cargo manipulation exist in pathophysiological contexts to lead to more or less virulence.^19^ ^,^ ^175^

The discovery and description of the interactions between EVs and host cells supposes the integration of new knowledge in the understanding of the phenomenon of parasitism. Furthermore, as cellular inducers, EVs immunomodulation has been widely proven. In fact, in biomedical application, the advantages offered using EVs as platforms for immunisation are being studied since they could represent stable carriers of various antigens, which would prevent the development of tolerance. However, aspects of logistics, formulation, safety, and effectiveness in suitable models cannot be ignored given still unpredictable responses.^175^
